# Concentration Dependent Influence of Lipopolysaccharides on Separation of Hoof Explants and Supernatant Lactic Acid Concentration in an *Ex Vivo/In Vitro* Laminitis Model

**DOI:** 10.1371/journal.pone.0143754

**Published:** 2015-11-24

**Authors:** Nicole Reisinger, Simone Schaumberger, Veronika Nagl, Sabine Hessenberger, Gerd Schatzmayr

**Affiliations:** BIOMIN Research Center, Tulln, Austria; INSERM, FRANCE

## Abstract

Laminitis is one of the most common diseases in horses. It is not only painful for the animal, but also has a significant financial impact on the equine industry. This multifactorial disease affects the connective tissue of the hoof. However, the pathogenesis of laminitis is still not fully understood. Endotoxins, also known as lipopolysaccharides (LPS), and bacterial exotoxins seem to play an important role during the development of laminitis. The aim of our study was to investigate the effect of increasing LPS concentrations (0, 2.5, 5, 10, and 100 μg/mL) on cell viability of isolated epidermal and dermal hoof cells as well as on the tissue integrity of hoof explants. Furthermore, glucose, acetic acid, lactic acid, and propionic acid concentrations in explant supernatants were measured to evaluate the energy metabolism in the hoof tissue. LPS did not exhibit cytotoxic effects on epidermal or dermal cells. Force required to separate LPS treated hoof explants decreased in a concentration dependent manner. Specifically, explants incubated with 10 and 100 μg/mL needed significantly less force to separate compared to control explants. Lactic acid concentrations were significantly decreased in explants incubated with 5, 10, or 100 μg/mL LPS, while glucose, acetic acid and propionic acid concentrations were unaffected by LPS treatment. Our study indicates that LPS has no cytotoxic effect on epidermal and dermal cells isolated from hoof tissue, but impairs integrity of hoof explants. In addition, LPS led to an alteration of the lactic acid production in the lamellar tissue. Since our data highlight that LPS can affect the integrity of the equine hoof tissue *in vitro*, endotoxins should be further explored for their contribution to facilitate the development of laminitis.

## Introduction

Laminitis, a disease which affects the lamellar tissue of the hoof, is painful for the animal. It has a significant impact on the horse industry [1]. The pathogenesis of laminitis is still not fully understood. As it has multifactorial etiology, several trigger factors are discussed, among them endotoxins, also known as lipopolysaccharides (LPS) [2–5] as well as bacterial exotoxins [6, 7]. Although systemic administration of LPS alone failed to induce laminitis, influences of endotoxins on hoof tissue could be demonstrated in several studies [8–10]. Furthermore, increased levels of LPS were measured in the serum of horses during laminitis experimentally induced with a carbohydrate or oligofructose overload [3, 11]. Also, therapy with anti-endotoxin serum helped to counteract laminitis [12], thus further strengthening the hypothesis that endotoxins play an important role to facilitate the development of laminitis.

*In vivo* studies have several limitations e.g. it is only possible to test a limited number of potential trigger factors in one trial. Hence, there is a high demand of alternatives to *in vivo* trials. *In vitro*/*ex vivo* testing with primary hoof cells or hoof tissue can provide an important tool to identify different factors influencing the pathogenesis of laminitis. However, systemic responses such as cytokine production or hormonal responses cannot be simulated in these models. Therefore, careful data interpretation is necessary and *in vivo* trials have to be performed to confirm the outcomes of these studies.

Although hoof cells have previously been isolated and cultivated from equine tissue [13–15], there is a lack of information about the effects of LPS on hoof cells. The first studies examining the effects of LPS on the lamellar hoof tissue became available just recently [6, 16]. At defined concentrations, LPS impaired the tissue integrity of hoof explants after 24 and 48 hours of incubation. These studies suggest that LPS can have a considerable influence on the equine hoof. Therefore, it is essential to study the mode of action of these harmful toxins.

Glucose metabolism in the lamellar tissue might play an important role in the pathogenesis of laminitis as it is essential to maintain lamellar integrity. The hoof has a comparably high demand for glucose. Previous studies showed that the structure of cultured hoof explants cannot be maintained without sufficient glucose supply [17, 18]. Changes of the glucose metabolism led to the separation of the basal epidermal cells from their basement membrane [17, 18], which is a process described to be typical for the pathogenesis for laminitis. A recent study by Medina-Torres *et al*. [19] showed that there was a significant decrease in the glucose concentration of lamellar dialysates during laminitis induced by oligofructose, probably due to increased glucose uptake and consumption. In general, studies about the glucose metabolism and glucose transporters in the lamellar tissue and their role during the pathogenesis of laminitis, especially insulin-induced laminitis, are controversial [20–22]. Beside glucose, lactate, an anion produced from lactic acid, represents an important energy source for the cells of the lamellar tissue [23]. However, there is no information available on the concentration of lactic acid or lactate produced by the lamellar tissue *in vitro*, nor on the influence of LPS on lactate production.

The aim of our study was to test the cytotoxic effects of increasing LPS concentrations on primary isolated epidermal and dermal hoof cells. In addition, the impact of increasing LPS concentrations on the lamellar tissue integrity was assessed *in vitro*. To elucidate the metabolism processes of the lamellar tissue during LPS exposure, the concentration of glucose and different acids of explant supernatants was measured. Results will provide new insights on the role of endotoxins during the pathogenesis of laminitis.

## Material and Methods

### Ethics statement

Equine hooves were obtained from a commercial slaughter house (Gumprecht, Austria). All horses were slaughtered for meat production and no animal was slaughtered specifically for the purpose of tissue collection.

### Animals

Forelimbs of eight adult horses were obtained from a local abattoir. There was no information given on age, gender, breed or history of the animals. Horses were stunned by use of a penetrating captive bolt. After stunning and subsequent exsanguination, forelimbs were disarticulated at the middle carpal joint. Hoof condition was assessed by visual inspection as well as macroscopic evaluation of the lamella. Only hooves with no sign of disease were further processed. The forelimbs were transported on ice to the laboratory. Time from death of the horses until arrival of the forelimbs at the laboratory did not exceed two hours.

### Preparation of explants

**Fig 1:**
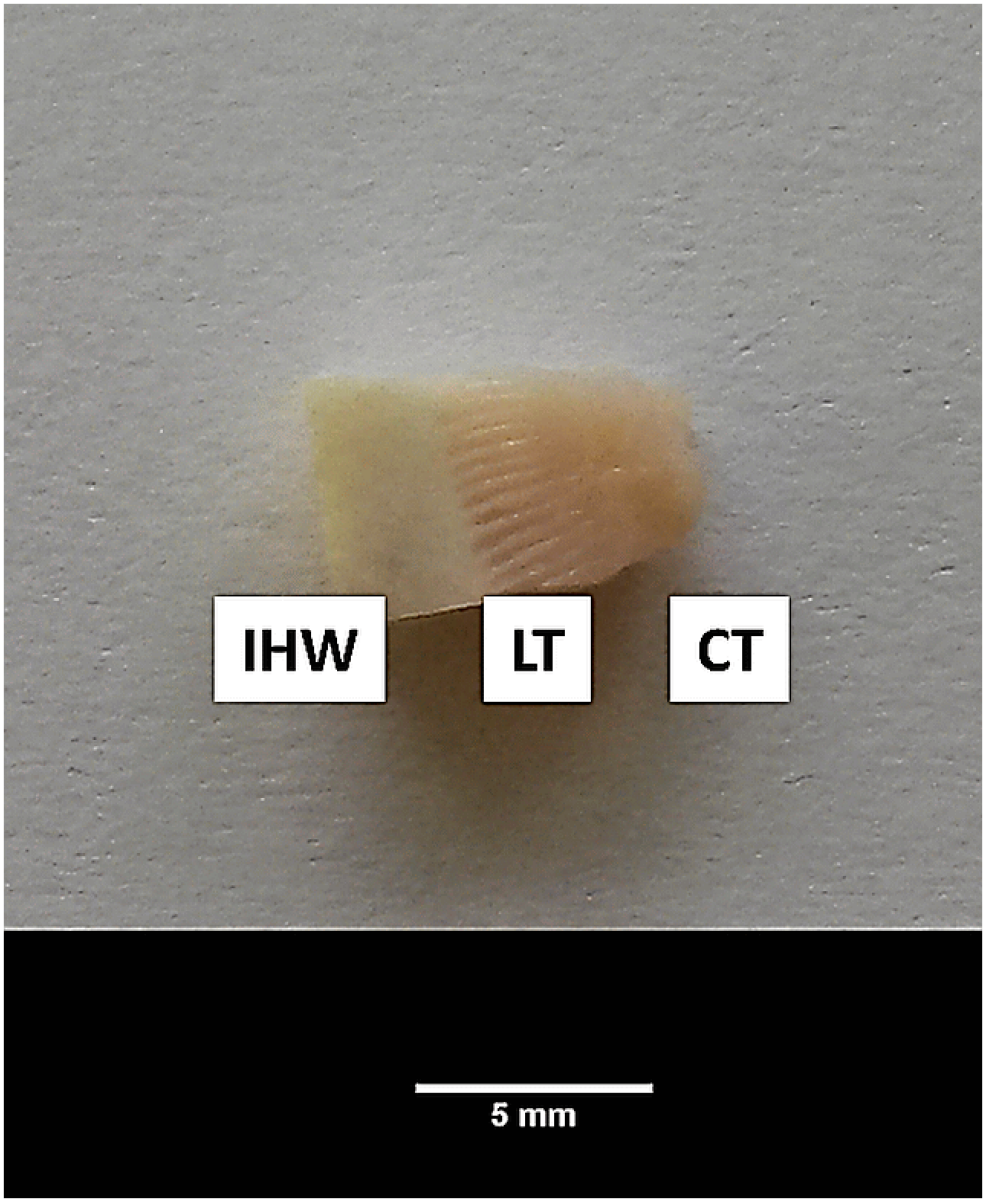
Schematic view of cultivated hoof explants. Hoof tissue explants used for cell isolation and separation testing containing inner hoof wall (IHW), lamellar tissue (LT) and connective tissue (CT).

Hooves were prepared and dissected as described by Pollitt [24]. Explants (Fig 1) were either used for cell isolation or were cultivated without further processing for separation testing.

### Isolation of cells from the epidermal and dermal hoof tissue

For cell isolation, explants were incubated with 50 μg/mL thermolysin (Sigma, Vienna, Austria) overnight at 37°C. Afterwards, dermis and epidermis were separated with two forceps, and pieces of the respective tissues were cultivated in 6-well plates (Eppendorf, Vienna, Austria). Three dermis pieces per well were incubated in plates coated with the Matrix Kit (Life technologies, Vienna, Austria) for outgrowth of dermal cells (DC). Three epidermis pieces per well were incubated in plates coated with Cultrex^®^ (Trevigen, Gaithersburg, MD, USA) for outgrowth of epidermal cells (EC) [25]. Gibco^®^ D-MEM (4.5 g/L glucose; Invitrogen, Vienna, Austria) was used as cultivation medium for dermal tissue pieces. RPMI 1640 medium (Sigma) supplemented with the Human Keratinocyte Growth Kit (Life Technologies, Vienna, Austria) was used as cultivation medium for epidermal tissue pieces. Both media were additionally supplemented with 10% fetal bovine serum (FBS; Life technologies), 0.025 M HEPES (Sigma) and an antibacterial/antimycotic solution (200 units/mL of penicillin, 200 μg/mL of streptomycin and 0.5 μg/mL of Fungizone^®^; Life technologies). Carefully, 500 μL medium was pipetted onto the tissue pieces to avoid flooding. After 24 hours, tissue pieces were attached to the plates and further 3 mL medium was added. Medium was changed twice a week. After 10 days, tissue pieces were removed leaving ECs and DCs in the wells.

### Culture, seeding and cytotoxicity tests of ECs and DCs

After two weeks, cells were subcultivated into T-25 flasks (Eppendorf) and subsequently in T-75 flasks (Eppendorf). After the first passage, a lower concentration of the antibacterial/antimycotic solution (100 units/mL of penicillin, 100 μg/mL of streptomycin and 0.25 μg/mL of Fungizone^®^) was used for both cell types. For ECs, the FBS concentration was additionally lowered to 5%. ECs and DCs were used until passage 6. For morphological fluorescence staining, epidermal cells and dermal cells were seeded at 1 x 10^4^ cells in chamber slides (Ibidi, Martinsried, Germany) coated with Cultrex^®^. For staining, cells were washed with HBSS (Life Technologies) for 15 min at 100 rpm at room temperature. Cells were fixed with 4% paraformaldehyde solution (Sigma) for 15 min at 37°C. Afterwards, cells were again washed three times with HBSS. Fluorescein labeled wheat germ agglutinin (Invitrogen) was added to the cells for 10 min at room temperature at 100 rpm at a dilution of 1:500. Cells were washed again with HBSS, and were visualized using an Eclipse E400 fluorescence microscope (Nikon, Vienna, Austria).

For cytotoxicity tests, cells were seeded in a 96-well plate (Eppendorf) with a density of 4 x 10^4^ cells per well and 200 μL culture medium per well. After 48 hours, LPS from *Escherichia coli* O55:B5 [0–100 mg/L] was added to the cells (3 wells per treatment) for 24 hours. Cell viability was tested with the water soluble tetrazolium (WST-1) reagent (Roche, Vienna, Austria) as described by the manufacturer. Four independent experiments were performed.

### Culture of explants, viability and lamellar separation testing

Explants were cultured with 1 mL medium at 37°C and 5% CO_2_ in quadruplicate in 24-well plates (Eppendorf) for 24 hours. D-MEM (4.5 g/L glucose) supplemented with 100 U/mL nystatin and 0.1 mg/mL gentamicin was used as culture medium (all Life technologies). Explants were cultured either with LPS [2.5–100 mg/L] or with culture medium only (negative control). Microscopic evaluation was used to determine bacterial or fungal contamination of explants. Contaminated explants were excluded from the results. For measurement of separation force of explants, a calibrated force transducer was used as described by Reisinger *et al*. [16]. Viability of explants was tested with the WST-1 assay [16].

### HPLC determination of glucose, acetic acid, lactic acid, and propionic acid concentrations of explant supernatants

Glucose, acetic acid, lactic acid, and propionic acid concentrations were determined in the explant supernatants by high performance liquid chromatography (HPLC). For sample preparation, Carrez solution I (1.06 g K_4_[Fe(CN)_6_]*3H_2_O in 10 mL distilled H_2_O) and Carrez solution II (2.88 g ZnSO_4_*7H_2_O in 10 mL distilled H_2_O) were added to the explant supernatants (diluted 1:5 with distilled water) and culture medium as control. After 10 minutes incubation at room temperature, supernatants were centrifuged at 16,600 x *g* for 30 minutes. Thereafter, supernatants were filtered through a 0.2 μm syringe filter and transferred into HPLC-vials. Samples were frozen at -20°C until analysis.

Analysis was carried out on a HPLC (Agilent 1100, Vienna, Austria), equipped with a refractive index detector set to 45°C. Isocratic elution was performed on an ICSep Ion-300 column (Transgenomic), using H_2_SO_4_ [5 mM] as mobile phase. The flow rate was set to 0.4 mL/min and the injection volume was 40 μL. Total time of analysis was 70 min. External standards were used for calibration and quantification.

### Statistical analysis

Statistical evaluation was performed with IBM SPSS Statistics 19 software. Data were tested for normal distribution with the Kolmogorov–Smirnov test. If data were normally distributed, ANOVA was performed with the Dunnett test as post-hoc test. If data were not normally distributed the Kruskal Wallis test was used as non-parametric test. Spearman correlation (two tailed) was used to calculate correlation between lactic acid concentration of supernatants and separation force of explants. Results were considered significant at p < 0.05.

## Results

### Cell isolation and culture

**Fig 2:**
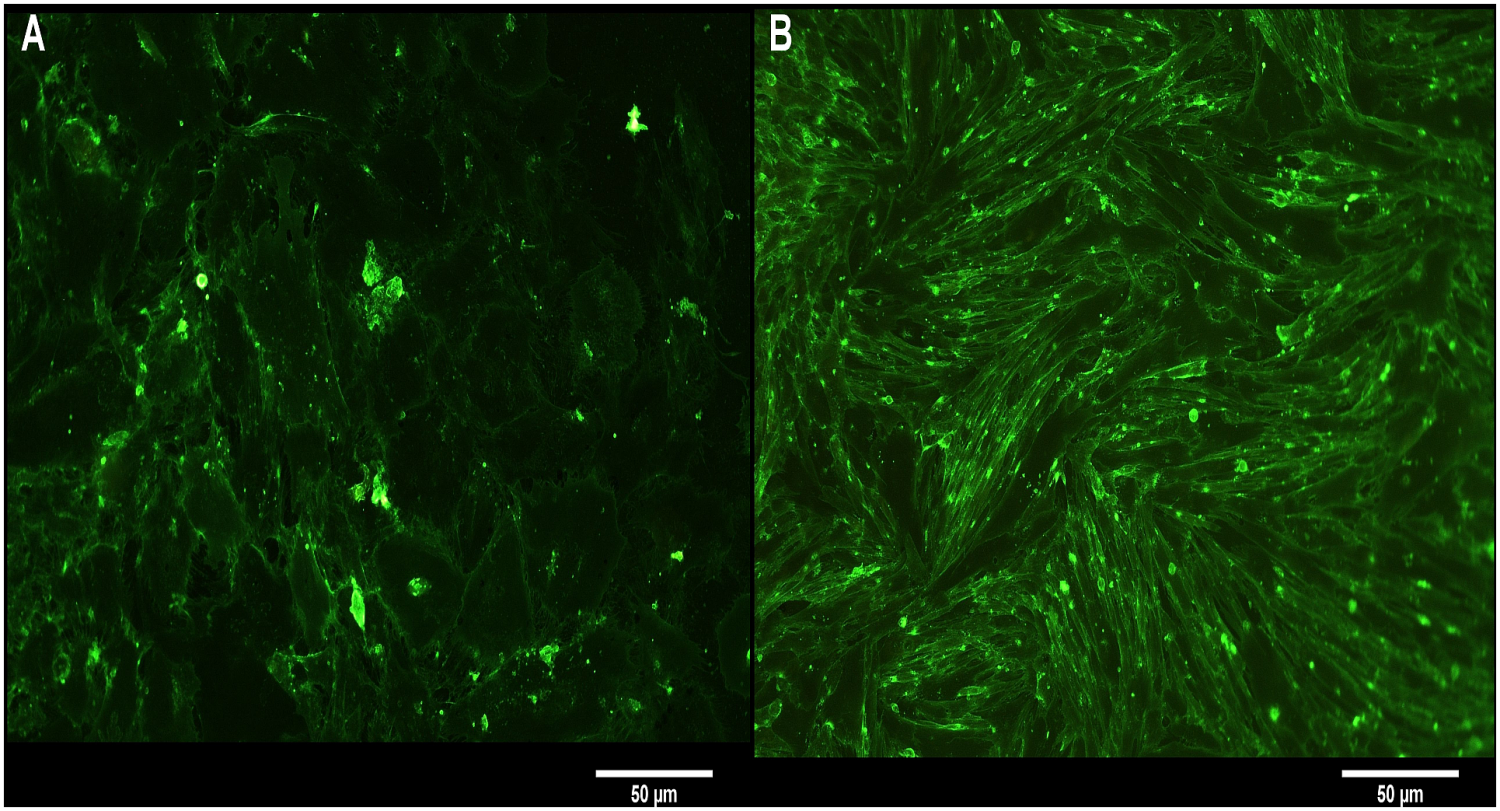
Morphology of primary isolated hoof cells. Epidermal (A) and dermal (B) cells stained with FITC conjugated wheat germ agglutinin.

Cells from two horses were used for cytotoxicity tests. Isolation and cultivation of epidermal and dermal cells was successful (Fig 2). Seven days after seeding of the tissue pieces, cells started to proliferate. After a total of two weeks, cells were confluent and could be subcultivated.

### Cytotoxicity tests of epidermal and dermal cells

**Fig 3:**
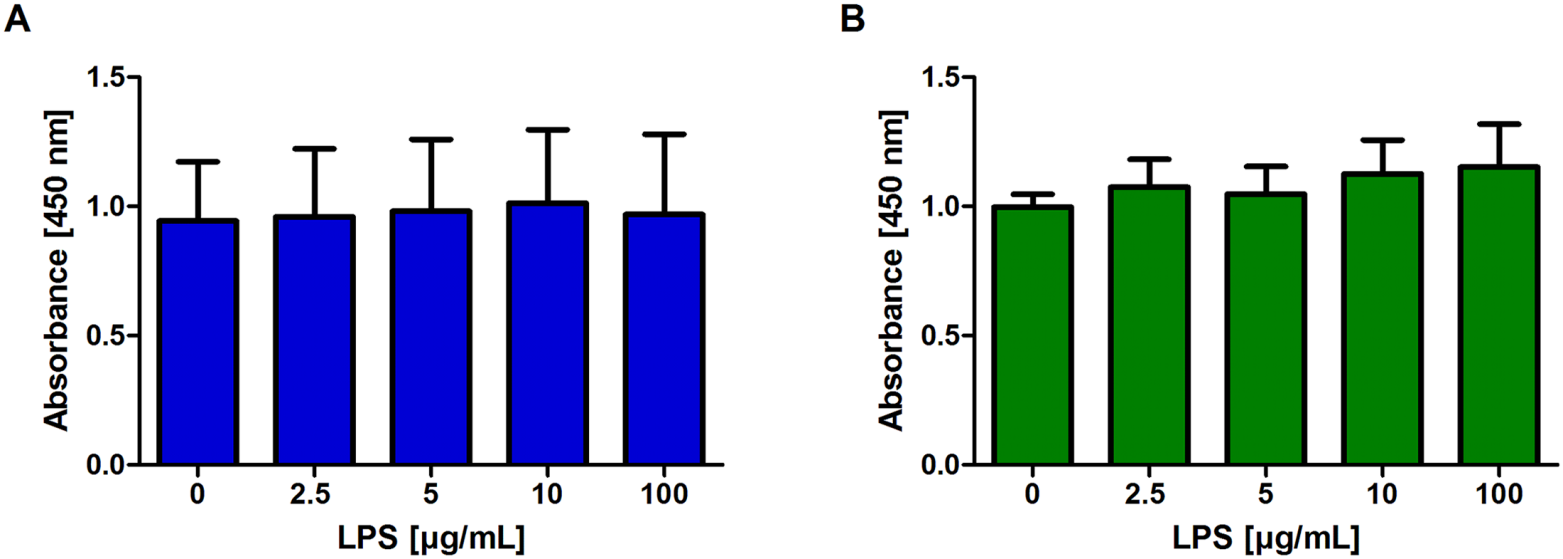
Viablity of primary isolated hoof cells incubated with LPS. Absorbance values (450 nm) of epidermal (A, blue) and dermal (B, green) cells incubated with medium or LPS (2.5–100 μg/mL) for 24 hours measured with the WST-1 assay (n = 4). Error bars display standard deviation.

Independent of the concentration, LPS had no influence on the viability of epidermal and dermal cells (Fig 3).

### Viability and separation force of explants

**Fig 4:**
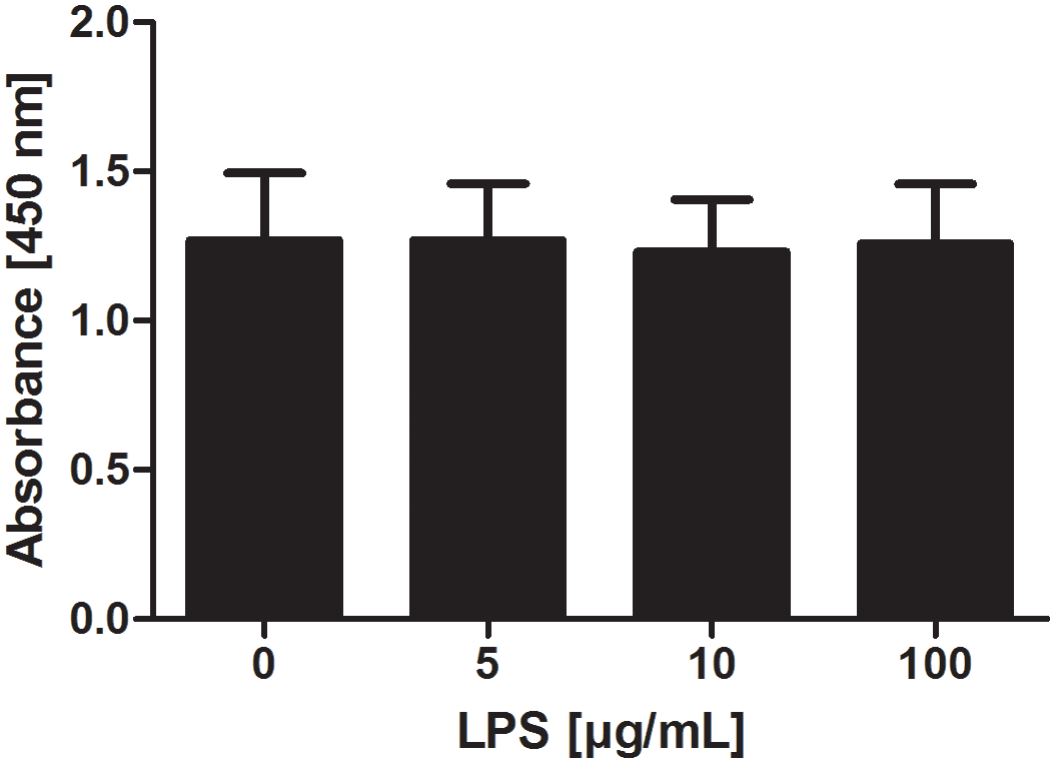
Viability of hoof explants incubated with LPS. Absorbance values (450 nm) of supernatants of explants incubated with medium or LPS (2.5–100 μg/mL) for 24 hours measured with the WST-1 assay (n = 3). Error bars display standard deviation.

There was no influence of tested LPS concentrations on viability of explants (Fig 4).

**Fig 5:**
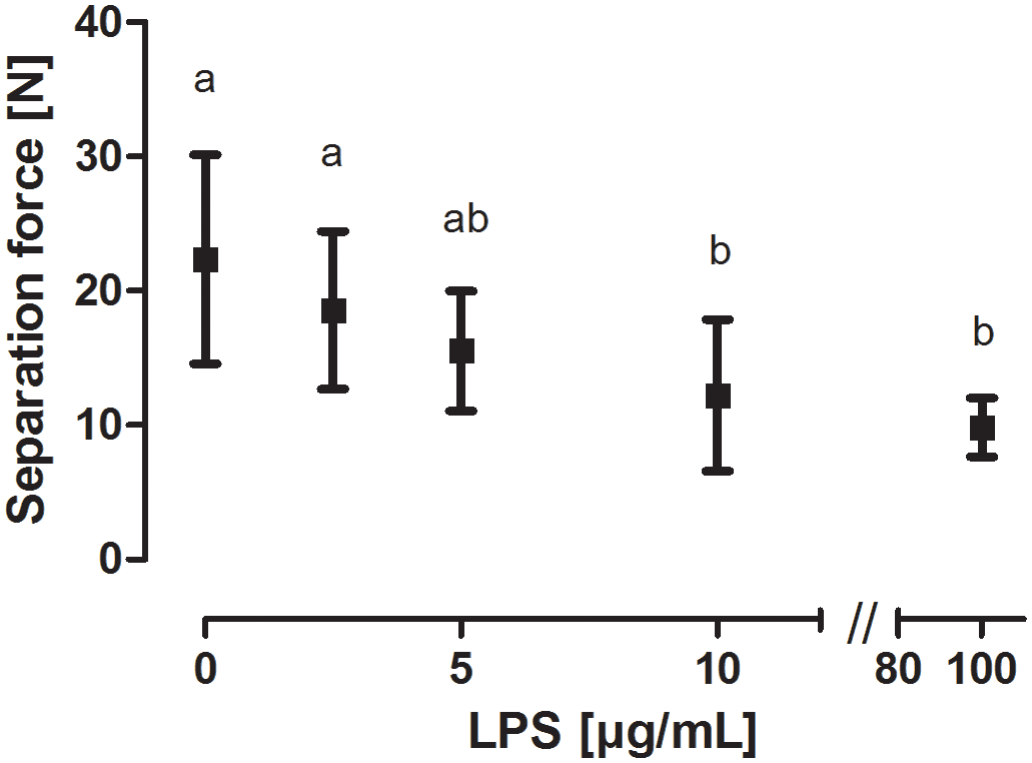
Separation force of hoof explants incubated with LPS. Separation force of explants incubated with medium or LPS (2.5–100 μg/mL) for 24 hours (n = 6 hooves). Error bars display standard deviation. ^ab^ Superscripts indicate significant difference p < 0.05.

Explants were tested for tissue integrity with a calibrated force transducer. The force needed to separate the explants was significantly reduced in samples incubated with 10 or 100 μg/mL LPS, compared to the controls (Fig 5).

### HPLC determination of glucose, acetic acid, lactic acid, and propionic acid concentrations of explant supernatants

**Fig 6:**
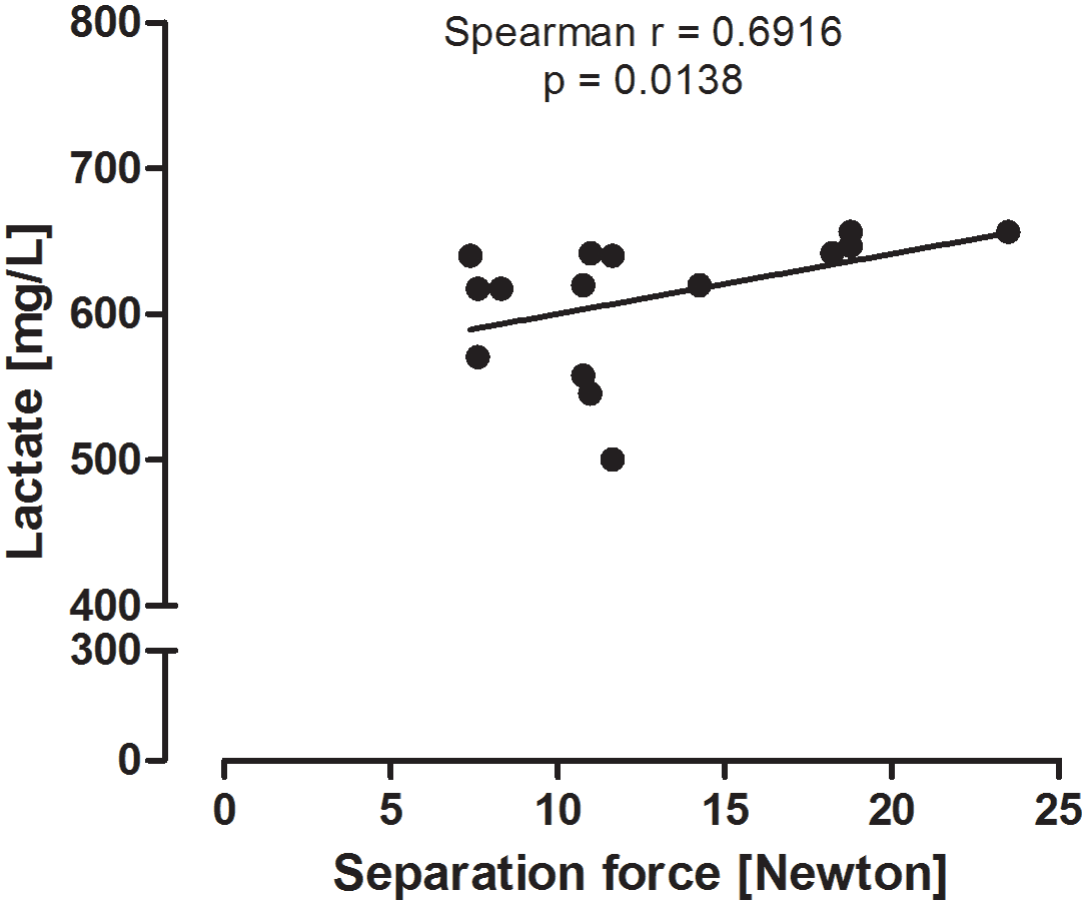
Correlation of lactic acid concentration in supernatants and separation force of explants. Correlation of lactic acid concentration in supernatants and separation force of explants incubated with medium or LPS [0–100 μg/mL] for 24 hours (n = 3 hooves).

After 24 hours of incubation with LPS, explant supernatants were analyzed for glucose, acetic acid, lactic acid, and propionic acid concentrations. Lactic acid concentrations were significantly decreased in supernatants of explants incubated with 5, 10, and 100 μg/mL LPS compared to explants incubated with 2.5 μg/mL LPS and control explants. In contrast, no influence of LPS on glucose, acetic acid, and propionic acid levels was observed (Table 1). In addition, there was a positive correlation between lactic acid concentration in explant supernatants and separation force of explants incubated with LPS (Fig 6).

**Table 1:** Glucose, acetic acid, lactic acid, and propionic acid concentration of supernatants of hoof explants incubated withLPS.

	Treatment of hoof explants						
LPS [μg/mL]	Culture medium	0	2.5	5	10	100	p-Value
**Glucose [mg/L]**	5,523	114.4	190.5	125.8	133.7	97.6	0.4708
**SEM**	-	14.6	76.5	24.3	19.1	17.9	
**Acetic acid [mg/L]**	0	21.42	21.75	26.85	25.25	26.00	0.9990
**SEM**	-	11.9	11.9	7.7	9.6	5.1	
**Lactic acid [mg/L]**	0	659.0 ^a^	614.0 ^a^	584.0 ^b^	589.0 ^b^	584.0 ^b^	**0.0324**
**SEM**	-	11.1	17.6	23.4	25.7	15.8	
**Propionic acid [mg/L]**	0	18.22	14.57	17.86	16.38	18.38	0.5110
**SEM**	-	1.4	2.4	1.7	2.1	1.7	

Glucose, acetic acid, lactic acid, and propionic acid concentration measured of culture medium and supernatant of explants (n = 12 explants) incubated with medium or LPS [0–100 μg/mL] for 24 hours.

^ab^ Superscripts indicate significant difference p < 0.05.

## Discussion

Although a lot of research has been conducted on the pathology of laminitis, it is still not fully understood and different trigger factors are discussed. Several studies have examined the involvement of endotoxins during intestinal disturbance and pathogenesis of laminitis. Determination of increased endotoxin levels in biological matrices was an important aspect in these experiments. For example, during colic events endotoxin concentrations of 90–220 pg/mL [26, 27] were measured in serum and up to 510 pg/mL in the peritoneal fluid [26], while during starch overload endotoxin concentrations were increased to 1.53 pg/mL [11]. Administration of 10 g/kg body weight oligofructose via nasogastric tube led to a concentration of 2.4 pg/mL LPS in plasma 8 hours after administration [3]. In contrast, endotoxin serum concentrations of 18 EU/mL (equivalent to 1,800 pg/mL) were measured in healthy pigs [28]. Higher endotoxin concentrations are likely to be due to the determination using an endotoxin assay with a recombinant factor C instead of the conventional *Limulus amebocyte* lysate (LAL) assay (highlighting the difficulties of measuring endotoxins in the blood with conventional methods).

One major problem in detection of endotoxins in the blood is the fast clearance of LPS by several proteins, e.g. LPS-binding protein or albumin [29]. In addition, sample collection time and storage conditions can have a significant influence on the recovery of LPS in the plasma [30]. Although it is common to use the LAL assay to measure endotoxins in the blood, it has to be mentioned that this assay is not intended for this purpose but rather for determination of endotoxins in simple matrices e.g. injection solutions. Several blood compounds, such as proteases, can cause interferences in this test system, leading to inaccurate quantification of endotoxins. It is therefore possible that higher endotoxin concentrations are released into the blood stream during colic events than measured with conventional assays. Together with the fact that a single administration of LPS is used as an isolated trigger factor, this might be the reason why comparably higher concentrations of LPS have to be used to cause negative effects on the hoof *in vitro*.

Our study showed that LPS (10 and 100 μg/mL) had a concentration dependent effect on the hoof lamellar integrity. There was no effect at the lower LPS concentrations (2.5 and 5 μg/mL), which might be influenced by the limited number of animals used for the experiments. Although laminitis has never been induced by LPS administration alone [10], there were several studies which highlight the negative effect of LPS on hoof health. Intravenous LPS infusion led to hoof discomfort with weight shifting, increased hoof temperature, decreased digital blood flow and lamellar perfusion as well as an increased heart rate and body temperature in horses [31]. Furthermore, pretreatment of horses with LPS (7.5 ng/kg body weight/hour *i*.*v*.*)* for 8 hours increased the incidence and severity of laminitis induced by oligofructose administration (5 g/kg body weight/hour via nasogastric tube) [10]. Tadros *et al*. [32] performed a similar study, but used a lower concentration of LPS (5 ng/kg body weight *i*.*v*.). LPS administration did not increase the number of horses developing laminitis in this experiment. Interestingly, if horses were divided into LPS responder and non-responder, effects could be seen on the inflammatory response in the blood, liver and lung of responder. These inflammatory processes are also likely to facilitate the development of laminitis. Overall, these studies emphasize the idea that endotoxins are not able to induce laminitis on their own, but can worsen the progress of the disease.

In addition to *in vivo* studies, cultivation of hoof explants *in vitro* seems to be a suitable model to test potential trigger factors and simulate events occurring during laminitis [18]. Studies using the explant model also tested the effects of endotoxins and exotoxins on lamellar separation. One study has already described that LPS (5–100 μg/mL) significantly increase the number of separated hoof explants after cultivation for 24 and 48 hours [16]. However, Reisinger *et al*. [16] measured the effect of only one LPS concentration [10 μg/mL] on separation force, and could demonstrate a significant reduction. Our data confirmed these findings and furthermore showed that there is a concentration dependent decrease of force needed to separate lamellar tissue when explants were incubated with LPS. This is in contrast to a study conducted by Mungall *et al*. [6], in which no concentration dependent effect of LPS was observed. Yet, there was no further information given on the LPS concentrations or LPS type used. However, this study also described separation of explants incubated with supernantant of *E*. *coli* cultures containing endotoxins.

One drawback of the study presented is that hoof explants are isolated from the whole organism and cannot mimic the complex processes occurring during laminitis including inflammation. During inflammation certain cytokines can be found in the serum samples of horses affected, and they can reach the hoof tissue in addition to endotoxins. The lack of cytokines might be an explanation why high LPS concentrations were necessary to induce lamellar separation. Nevertheless, the separation of the tissue is a severe stage of tissue destruction and it cannot be excluded that already lower concentrations had effects on the lamellar structure. Further experiments focusing on processes happening before lamellar separation are essential.

Independent of the LPS concentration [0, 2.5, 5, 10, and 100 μg/mL], there was no cytotoxic effect on cells isolated from the dermis and epidermis of the equine hoof. On the one hand, previous studies showed no cytotoxic effect of LPS on equine macrophage cell line (eCAS) [33] or astrocytes cell line [34] and even demonstrated an increased proliferation of human fibroblasts and keratinocytes after LPS treatment [35]. On the other hand, it was reported that LPS inhibited the cell growth of mesenchymal cell lines, epithelial cells and significantly decreased the viability of mouse macrophage cell line (RAW 264.7) [36]. Hence, cytotoxic effects of LPS seem to be quite variable and most likely depend on cell type and LPS concentration used. However, in our study LPS did not show any effect on cells isolated from the equine hoof. We can therefore exclude that effects of LPS on lamellar tissue integrity were due to cytotoxicity of LPS. Furthermore, there was no effect of LPS on viability of cultured explants.

There are several potential mechanisms to explain how LPS possibly triggers the development of laminitis, e.g. activation of matrix metalloproteinases [37, 38] or onset of oxidative stress and inflammation cascades [39]. In addition, LPS is discussed to alter glucose metabolism. Glucose supply is essential for maintenance of hoof structure in the explant model. Explants incubated in saline containing glucose separated after 2 days [18], while separation force of explants incubated with D-MEM medium without glucose already decreased significantly after 12 hours [17]. To ensure sufficient glucose supply in our study, all explants were cultivated in medium containing high glucose concentrations [4.5 g/L]. Despite this fact, explants incubated with LPS separated. Surprisingly, we could not observe an increased glucose consumption when explants were incubated with LPS. Nevertheless, there was a great variation in the glucose concentration of explants supernatants, which might had an influence on obtained results. For further studies, it will be important to examine not only the glucose uptake but also glucose consumption.

To gain a broader picture of the energy metabolism, explant supernatants were additionally analyzed for levels of acetic acid, lactic acid, and propionic acid. Lactic acid concentration was significantly decreased in supernatants of explants incubated with LPS (5, 10, and 100 μg/mL). This decrease was concentration dependent and positively correlated with decreased separation force. In contrast, LPS treatment did not affect acetic and propionic acid levels, which might have indicated a more extensive alteration of the energy metabolism.

Lactate is described as an important product of the glucose metabolism in the lamellar tissue [23]. It can be used for ATP production, as it can enter the citric acid cycle *via* pyruvate. Increased lactate concentrations in the blood have been associated with development of laminitis [40]. Yet, injection of lactic acid into the digital arteries did not cause any clinical sign of laminitis [23]. Also, addition of 20 μmol/L lactic acid to cultivation media did not induce lamellar separation in explants incubated for 2 days [18]. Based on the results of Patan *et al*. [41], who noted an increase of lactate concentration and glucose consumption during limb perfusion with autologous blood containing LPS [80 ng/mL], we originally expected an increase in lactic acid in explants exposed to LPS. However, it is difficult to compare our findings to the mentioned study, as a different type of model was used. Still, both studies showed that there are changes in the lactate and lactic acid concentration during LPS administration, indicating a principal alteration of the glucose metabolism by endotoxins.

Tissue microdialysis [42, 43], which was used to measure lamellar energy metabolism during development of laminitis in the oligofructose model, showed an alteration of the glucose consumption, but could not detect a significant reduction or increase of lactate in lamellar dialysates. As lamellar energy metabolism seems to be a complex mechanism and our findings are preliminary data, they have to be confirmed by further studies.

In addition, it will be necessary to measure pyruvate concentration in the explant supernatant to have a complex picture about the energy metabolism. Furthermore, it will be interesting to evaluate the influence of LPS on glucose transport proteins and lactate production in the hoof.

### Conclusion

Our study indicates that LPS has no cytotoxic effect on dermal and epidermal cells isolated from the hoof but exhibits a negative influence on lamellar tissue integrity of cultured hoof explants cultured. Glucose metabolism and lactic acid formation in the lamellar tissue might play an important role during development of laminitis and should be further investigated. Endotoxins may not be capable to induce laminitis on their own, but seem to play an important role to facilitate the development of laminitis. Endotoxins should be further explored for their contribution during the pathogenesis of laminitis.

## Supporting Information

S1 File Average values of viabilty of primary cells and viability and separation foce of hoof explants. Average values of viability of epidermal and dermal cells incubated with LPS [0–100 μg/mL). Average values of viability and separation force of explants incubated with LPS [0–100 μg/mL).
(XLSX)
